# Impact of carbapenem-resistant *Klebsiella pneumoniae* infection on gut microbiota and host immunity: a case-control study

**DOI:** 10.1128/spectrum.02975-25

**Published:** 2025-11-20

**Authors:** Wenwen Ding, Zongxin Ling, Xia Liu, Jingchen Zhang, Yiwen Cheng, Zhangcheng Zhu, Lingbin Wu, Xiaocui Xu, Yongtao Gao, Xiaogang Hu

**Affiliations:** 1 Department of Anesthesiology, Affiliated Hospital of Nantong University, Medical School of Nantong University, Jiangsu, China; 2 State Key Laboratory for Diagnosis and Treatment of Infectious Diseases, National Clinical Research Center for Infectious Diseases, China-Singapore Belt and Road Joint Laboratory on Infection Research and Drug Development, National Medical Center for Infectious Diseases, Collaborative Innovation Center for Diagnosis and Treatment of Infectious Diseases, The First Affiliated Hospital, Zhejiang University School of Medicine, Hangzhou, Zhejiang, China; 3 Yuhang Institute for Collaborative Innovation and Translational Research in Life Sciences and Technology, Hangzhou, Zhejiang, China; 4 Department of Intensive Care Unit, The First Affiliated Hospital, Zhejiang University School of Medicine, Hangzhou, Zhejiang, China; 5 Department of Preventive Medicine, School of Public Health and Management, Wenzhou Medical University, Wenzhou, Zhejiang, China; 6 Department of Laboratory Medicine, Lishui Second People’s Hospital, Lishui, Zhejiang, China; 7 Department of Intensive Care Unit, Lishui Second People’s Hospital, Lishui, Zhejiang, China; University of Arkansas for Medical Sciences, Little Rock, Arkansas, USA

**Keywords:** enterotype, gut microbiota, *Klebsiella pneumoniae*, inflammation, multiplex immunoassays, sequencing

## Abstract

Carbapenem-resistant *Klebsiella pneumoniae* (CRKP) represents a critical global health threat with limited treatment options. While the gut microbiota is a reservoir for opportunistic pathogens and a regulator of host immunity, the reciprocal impact of systemic CRKP infection on gut microbial ecology and immune responses remains poorly defined. In a prospective case-control study, 38 patients with confirmed CRKP infection and 38 matched hospitalized controls without CRKP were enrolled. Fecal samples underwent 16S rRNA gene sequencing to characterize microbial profiles, and serum cytokine levels were quantified using multiplex immunoassays. CRKP infection was associated with significantly reduced microbial diversity and a distinct shift in community structure, characterized by depletion of beneficial commensals (*Bacteroides*, *Faecalibacterium*, *Roseburia*) and enrichment of pathobionts (*Klebsiella*, *Enterococcus*). Enterotype analysis revealed a predominance of a *Klebsiella*/*Enterococcus*-dominated enterotype in CRKP patients. Functional predictions indicated impaired carbohydrate and butyrate metabolism alongside increased virulence- and resistance-associated pathways. Systemically, patients exhibited elevated pro-inflammatory cytokines (IL-6, TNF-α, IFN-γ) and chemokines (IP-10, MCP-1, RANTES). Correlation analyses linked opportunistic taxa with heightened inflammatory markers, while beneficial short-chain fatty acid producers showed inverse associations. Systemic CRKP infection is associated with profound gut dysbiosis and a hyper-inflammatory immune response. The strong microbiota–immune correlations suggest that the gut microbiota may serve as a biomarker and a potential therapeutic target for mitigating CRKP-associated immune dysfunction, though the directional relationship (cause vs. consequence) between dysbiosis and CRKP infection remains to be elucidated.

**IMPORTANCE:**

Carbapenem-resistant *Klebsiella pneumoniae* (CRKP) is a critical global threat with limited therapeutic options. This study reveals that systemic CRKP infection is associated with profound gut dysbiosis—characterized by loss of beneficial commensals (e.g., *Faecalibacterium*) and expansion of pathobionts (e.g., *Klebsiella*, *Enterococcus*)—as well as a hyperinflammatory immune response. We demonstrate strong correlations between specific microbial taxa and host cytokines, suggesting that the gut microbiome may hold potential as a biomarker and therapeutic target. These findings enhance our understanding of host-microbe interactions in CRKP infection and support the exploration of microbiota-based therapies. However, further studies, including longitudinal and animal models, are needed to clarify whether gut dysbiosis directly influences CRKP outcomes or is a secondary consequence.

## INTRODUCTION

Carbapenem-resistant *Klebsiella pneumoniae* (CRKP) has emerged as one of the most important multidrug-resistant pathogens worldwide, causing severe infections such as pneumonia, bloodstream infections, and urinary tract infections (1). CRKP exhibits extensive resistance not only to carbapenems (e.g., imipenem, meropenem, ertapenem) but also to other last-line antibiotics including fluoroquinolones, aminoglycosides, and third-generation cephalosporins, leading to limited therapeutic options and high mortality rates, particularly among immunocompromised or critically ill patients (2). Surveillance data from China indicate a sharp increase in CRKP rates, from 7.6% in 2010 to 21.2% in 2018 (3). Given its escalating prevalence and severe clinical outcomes, the World Health Organization has designated CRKP as a priority pathogen requiring urgent development of innovative therapeutic strategies (4).

Beyond its role as a systemic pathogen, the gut serves as a key reservoir for CRKP colonization and dissemination (5). A healthy gut microbiota confers colonization resistance (CR) against invading pathogens (6). Specific commensals, including *Lactiplantibacillus plantarum* and *Bifidobacterium longum*, have been shown to facilitate CRKP clearance (7, 8). These beneficial commensals can enhance CR through competitive exclusion for nutrients and adhesion sites, production of antimicrobial compounds such as bacteriocins and organic acids, and modulation of host immune responses. However, factors such as broad-spectrum antibiotic use or underlying host conditions can disrupt microbial homeostasis, leading to dysbiosis, impairing CR, and facilitating CRKP overgrowth and subsequent infection. This process is closely linked to immune dysfunction: immunosuppressed patients are more susceptible to CRKP infection and tend to experience worse clinical outcomes. This susceptibility may be exacerbated by pre-existing or treatment-induced gut dysbiosis, which can further impair immune function and promote CRKP persistence and transmission (9). Additionally, systemic CRKP infection is often accompanied by strong inflammatory responses, which may, in turn, affect the gut environment. It has been proposed that this may create a potential cycle of dysbiosis and immune dysregulation that could promote the persistence of multidrug-resistant pathogens such as *K. pneumoniae* and *Enterococcus* species (10, 11). Deciphering the specific changes in the gut microbial ecosystem and systemic immunity during CRKP infection is therefore critical.

Despite these insights, most prior studies have focused predominantly on bacterial genetics and epidemiological trends, with limited attention paid to the dynamic interactions between the host microbiota and immune system during active CRKP infection. Consequently, the specific impacts of systemic CRKP infection, including infections at extraintestinal sites such as the bloodstream, lungs, or urinary tract, on gut microbiota structure and systemic immunity remain poorly characterized, hindering a comprehensive understanding of its pathogenesis and the development of microbiota-targeted adjuvant therapies. To address this gap, we conducted a prospective case-control study integrating 16S rRNA gene sequencing of gut microbiota with multiplex immunoassays for systemic cytokine profiling in CRKP-infected patients and matched hospitalized controls. Our study aims to (i) characterize the structural and functional alterations in the gut microbiota during CRKP infection (ii); evaluate concomitant changes in host systemic immune responses; and (iii) explore the correlations between specific microbial taxa and inflammatory markers. These findings are expected to provide mechanistic insights into host-microbe crosstalk during CRKP infection and support the future development of microbiome-based interventions to combat this formidable pathogen.

## MATERIALS AND METHODS

### Participant enrollment

This prospective case-control study was conducted at the Department of Intensive Care Unit (ICU) of The First Affiliated Hospital, Zhejiang University School of Medicine, Hangzhou, China, between September 2024 and March 2025. Adult patients (≥18 years) diagnosed with a CRKP infection were enrolled. CRKP infection was confirmed through both clinical and microbiological criteria. Participants were required to show clinical signs and symptoms of infection, such as fever, leukocytosis, hypoxemia, or hypotension. Additionally, *K. pneumoniae* was isolated from at least one sample (e.g., feces, blood, bronchoalveolar lavage fluid, or urine) that demonstrated resistance to at least one carbapenem antibiotic (imipenem, meropenem, or ertapenem), as determined by phenotypic testing in the clinical microbiology laboratory. Controls (Con) were selected from hospitalized patients during the same period, matched to the case group by age (±5 years), sex, and primary underlying disease. The majority of Con were patients with solid tumors (e.g., lung cancer, liver cancer, gastric cancer) who were admitted for routine chemotherapy or supportive care. These patients had no clinical or microbiological evidence of active infection and no history of CRKP infection within the past 90 days. Exclusion criteria for both groups included the following: (i) use of probiotics, prebiotics, or synbiotics within 2 weeks before enrollment; (ii) previous fecal microbiota transplantation (FMT); (iii) diagnosis of active inflammatory bowel disease; and (iv) pregnancy.

### Sample collection and processing

For each participant, fecal and peripheral blood samples were collected within 48 hours of enrollment. Fresh fecal samples were collected in sterile containers, immediately placed on ice, and transferred to a −80°C freezer within 2 hours of collection for long-term storage prior to DNA extraction. Serum samples were obtained from fasting participants in the early morning. The serum was separated by centrifugation at 3,000 rpm for 10 minutes, aliquoted, and stored at −80°C for cytokine analysis.

### Gut microbiota analysis via 16S rRNA gene sequencing

Total bacterial genomic DNA was extracted from 200 mg fecal samples using QIAamp DNA Stool Mini Kit (QIAGEN, Hilden, Germany) according to the manufacturer’s instructions. The quantity and quality of the extracted DNA were assessed using a NanoDrop ND-1000 spectrophotometer (Thermo Fisher Scientific, Boston, MA, USA) and 1% agarose gel electrophoresis, respectively. The V3-V4 hypervariable region of the 16S rRNA gene was amplified by PCR. The resulting amplicons were purified, quantified, and sequenced on a NovaSeq 6000 system (Illumina, USA). Negative DNA extraction controls (lysis buffer and kit reagents only) were amplified and sequenced as contamination controls (12–17). Library construction and sequencing were carried out at Hangzhou KaiTai Bio-lab.

### Bioinformatic analysis

Raw sequencing data were processed using the QIIME 2 (Quantitative Insights into Microbial Ecology 2) pipeline (version 2023.7) with default parameters (12–16, 18–20). DADA2 was used for sequence quality control, denoising, and merging to generate amplicon sequence variants (ASVs). Taxonomic assignment of ASVs was performed against the SILVA database (Release 138, http://www.arb-silva.de) using a pre-trained naïve Bayes classifier (21, 22). To minimize artifacts, ASVs accounting for fewer than 0.005% of total sequences were excluded, and sequences classified as Eukarya, Archaea, Mitochondria, Chloroplasts, or Cyanobacteria were removed to retain only bacterial profiles for downstream analysis (23). Bacterial α-diversity was assessed using the Shannon and Simpson indices, along with richness indices including ACE, Chao1, and observed species, based on the ASV table generated by DADA2. For β-diversity analysis, Bray-Curtis, Jaccard, unweighted, and weighted UniFrac distance metrics were computed. These β-diversity distances were visualized via principal coordinate analysis (PCoA) (24). Permutational multivariate analysis of variance (PERMANOVA) was used to test for significant differences in community structure. Taxa with low abundance, defined as those appearing in fewer than 50% of samples, were excluded from subsequent analysis. Enterotype classification was performed to group samples based on microbiome similarity, using genus-level abundance data that had been rarefied. This analysis was conducted using the OECloud tools (https://cloud.oebiotech.com). To identify differentially abundant taxa, linear discriminant analysis (LDA) effect size (LEfSe) was employed to identify bacterial taxa that were differentially abundant between the CRKP and Con groups, using an LDA score threshold of >3.0 and a false discovery rate (FDR)-corrected *P* < 0.05 (25). Microbial associations at the genus level were inferred using the sparse compositional correlation (SparCC) algorithm, which accounts for compositional bias. For functional prediction, predictive analysis of microbial community functions was performed using PiCRUSt2 version 2.3.0, which compares ASV-derived functional predictions to the Kyoto Encyclopedia of Genes and Genomes (KEGG) database. Microbial functions were categorized according to KEGG pathways at levels 1–3, and comparisons were made using STAMP (26, 27).

### Host immune profiling

Serum levels of key pro-inflammatory and anti-inflammatory cytokines/chemokines were measured using the Bio-Plex Pro Human Cytokine 27-plex assay kit (M500KCAF0Y, Bio-Rad, Hercules, CA, USA), according to the manufacturer’s instructions (12–16, 28, 29). This multiplex assay utilizes Luminex xMAP technology, allowing the simultaneous measurement of 27 immune markers, including 16 cytokines, 6 chemokines, and 5 growth factors. The analysis was performed on the Luminex 200 system (Bio-Rad), where fluorescence values corresponding to each analyte were recorded. Prior to analysis, serum samples were diluted fourfold with the sample diluent buffer. Cytokine concentrations were calculated from standard curves and expressed in pg/mL, using Bio-Plex Manager v5.0 software. The assay demonstrated high reproducibility, with coefficients of variation ranging from 5% to 8%. Quality control measures included the validation of standard curves, checks for dynamic range, and rigorous controls to confirm assay specificity. Outlier values were flagged for further scrutiny, and cytokine concentrations falling below the limit of detection (LOD) were assigned half the LOD value, ensuring the integrity of the data set. These quality assurance protocols were critical to maintain both the accuracy and reliability of the results.

### Statistical analysis

Statistical tests were chosen based on the data type. For continuous variables—such as α-diversity indices, taxonomic abundance, and cytokine levels—comparisons were made using either White’s nonparametric *t*-test, independent *t*-test, or the Mann-Whitney *U*-test, depending on the data distribution. Categorical variables were analyzed using Pearson’s chi-square test or Fisher’s exact test. To explore relationships between microbial abundances and cytokine levels, Spearman’s rank correlation was used. All statistical analyses were performed with SPSS v24.0 (SPSS Inc., Chicago, IL) and STAMP v2.1.3 software, with graphical representations generated via R packages and GraphPad Prism v6.0. For assessing the discriminative power of key functional taxa, random forest classification was applied, with the importance of variables determined by mean decrease Gini. Higher mean decrease Gini values indicate greater importance of a particular genus. The model’s predictive accuracy was evaluated using receiver operating characteristic (ROC) curves and area under the curve (AUC) analysis, carried out through OECloud tools. All tests were two-sided, and *P*-values were adjusted for multiple comparisons using the Benjamini-Hochberg method to control FDR, with an FDR threshold of <0.05 considered statistically significant.

## RESULTS

### Patient demographics and clinical characteristics

A total of 76 participants were enrolled: 38 in the CRKP group and 38 in the matched Con group. The baseline demographic and clinical characteristics of both cohorts are summarized in Table 1. There were no significant differences in age, sex, body mass index, or the prevalence of major comorbidities such as diabetes mellitus, chronic kidney disease, solid tumors, and hematologic malignancy between the two groups, indicating successful matching. As expected, clinical signs of infection and systemic inflammatory markers were significantly more severe in the CRKP group. These included higher body temperature, elevated white blood cell count, and increased levels of C-reactive protein, procalcitonin, and ESR (all *P* < 0.05). Furthermore, all CRKP isolates exhibited resistance to carbapenems, with high rates of resistance to other antibiotic classes, including fluoroquinolones (76.3%), aminoglycosides (84.2%), and third-generation cephalosporins (100%). Multidrug resistance was observed in 92.1% of the CRKP isolates.

**TABLE 1:** Baseline demographic and clinical characteristics of the study participants^*a*^

Characteristic	CRKP (*n* = 38)	Con (*n* = 38)	*P*-value
Age, years	62.4 ± 10.7	61.1 ± 9.8	0.584
Sex, male	22 (57.9%)	20 (52.6%)	0.648
BMI, kg/m²	23.6 ± 3.2	24.1 ± 2.9	0.472
Comorbidities			
Diabetes mellitus	11 (28.9%)	9 (23.7%)	0.602
Chronic kidney disease	6 (15.8%)	5 (13.2%)	0.746
Solid tumor	13 (34.2%)	10 (26.3%)	0.456
Clinical signs			
Body temperature, °C	38.7 ± 0.9	36.9 ± 0.4	<0.001
Hypotension	15 (39.5%)	2 (5.3%)	<0.001
Hypoxemia	17 (44.7%)	3 (7.9%)	<0.001
Laboratory markers			
WBC, ×10⁹/L	14.6 ± 3.8	6.8 ± 1.5	<0.001
CRP, mg/L	89.5 ± 28.4	5.2 ± 2.1	<0.001
PCT, ng/mL	3.6 ± 1.5	0.08 ± 0.03	<0.001
ESR, mm/h	48.2 ± 16.3	12.5 ± 5.8	<0.001

^*a*^ BMI, body mass index; CRP, C-reactive protein; ESR, erythrocyte sedimentation rate; PCT, procalcitonin; WBC, white blood cell count. Data are presented as mean ± standard deviation or *n* (%).

### Changed fecal microbiota structure in CRKP infection

**Fig 1:**
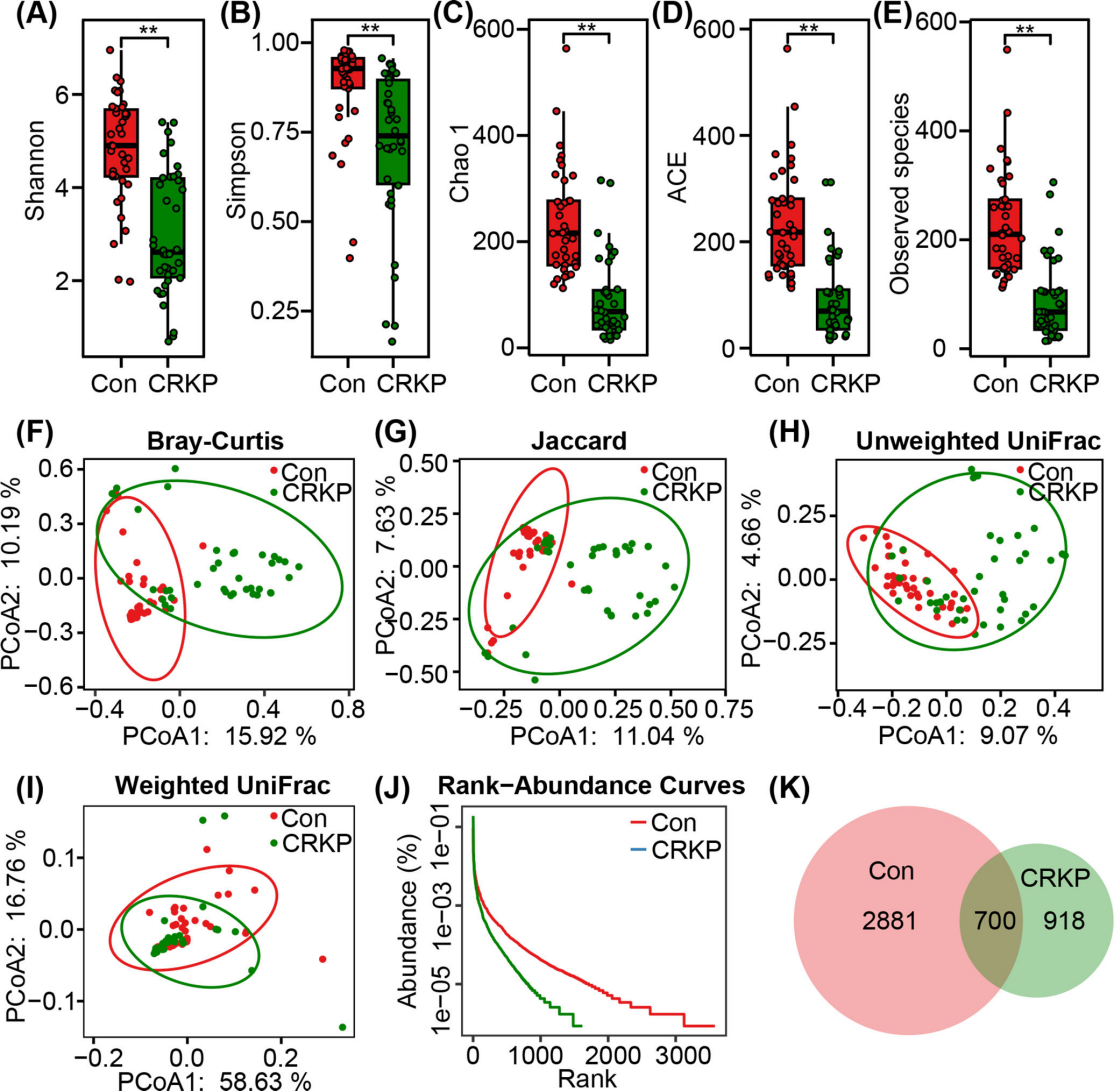
Structural alterations in the gut microbiota of CRKP-infected patients. α-diversity was assessed using the Shannon (**A**) and Simpson (**B**) indices, and species richness was evaluated via ACE (**C**), Chao1 (**D**), and observed species (**E**) indices. Comparisons were made between CRKP-infected patients and controls (Con). Data are presented as mean ± standard deviation. Statistical significance was determined using unpaired two-tailed *t*-tests; **P < 0.01. β-Diversity was visualized using PCoA based on Bray-Curtis (**F**), Jaccard (**G**), unweighted UniFrac (**H**), and weighted UniFrac (**I**) distance metrics, revealing significant separation between the CRKP and Con groups (PERMANOVA, *P* < 0.001 for all metrics). Each point represents an individual sample. The Venn diagram shows the number of unique and shared ASVs between groups (**J**). Rank-abundance curves further illustrate differences in species evenness and richness between CRKP patients and controls (**K**).

A total of 4,003,438 high-quality sequencing reads were obtained, including 2,275,687 reads from Con and 1,727,751 from CRKP patients, with an average of 52,676 reads per sample. For downstream analyses, data were rarefied to 30,777 reads per sample to ensure even sequencing depth. This normalization identified 4,499 bacterial ASVs across all groups, with a Good’s coverage estimate of 99.96%, confirming near-complete representation of the fecal microbiota. To assess the impact of CRKP on the overall structure of the gut microbiota, we first evaluated bacterial α-diversity, which reflects species richness and evenness within individual samples. The CRKP group exhibited significantly lower microbial diversity (Shannon and Simpson) and richness (Chao 1, ACE, and Observed species) compared to the Con group (Fig. 1A through E). This indicates a significant contraction of the microbial ecosystem in patients with CRKP infection. We next examined beta diversity to assess differences in microbial community structure between the groups. PCoA based on multiple distance metrics, including Bray-Curtis, Jaccard, unweighted UniFrac, and weighted UniFrac, revealed clear and statistically significant separation between the CRKP and Con groups (PERMANOVA, *P* < 0.001 for all metrics) (Fig. 1F through I). Additionally, rank-abundance curves showed a less steep and shorter tail in the CRKP group compared to controls (Fig. 1J), further supporting the reduction in species richness and evenness. Furthermore, the Venn diagram showed a lower number of unique bacterial phylotypes in the CRKP group (918 ASVs) compared to Con (2,881 ASVs) (Fig. 1J). Together, these findings demonstrate that CRKP infection is linked to significant disruptions in gut microbial ecology, characterized by loss of diversity and altered community structure.

### Altered fecal microbiota composition in CRKP infection

**Fig 2:**
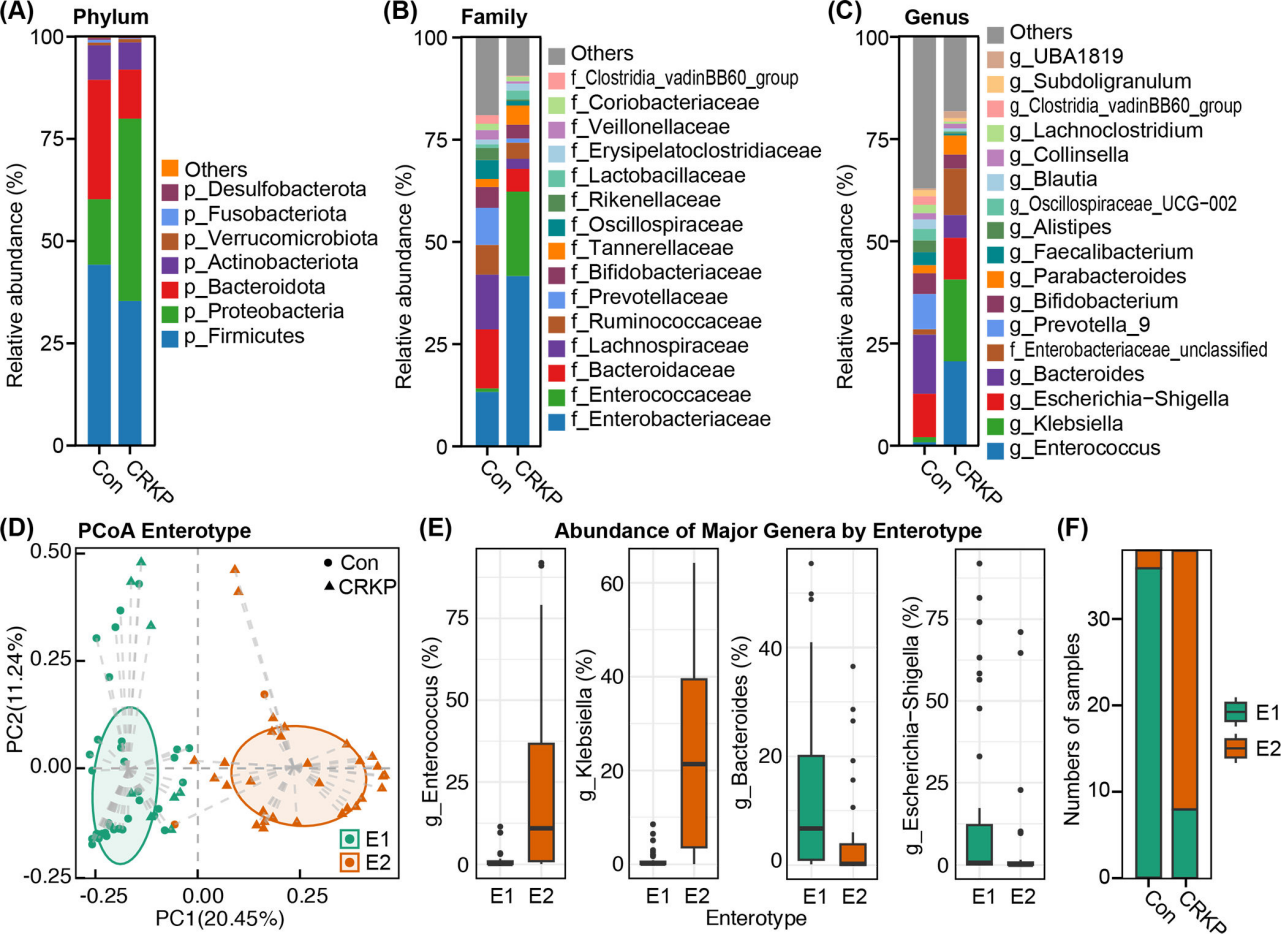
Gut microbial community composition and enterotype distribution in CRKP-infected patients and controls. Relative abundance of dominant bacterial taxa at the phylum (**A**), family (**B**), and genus (**C**) levels. (**D**) PCoA based on genus-level profiles, illustrating the separation of samples into two distinct enterotypes: E1 (*Bacteroides*-dominated) and E2 (*Klebsiella*-dominated). (**E**) Abundances of signature genera (*Enterococcus*, *Escherichia-Shigella*, *Klebsiella*, and *Bacteroides*) within each enterotype. (**F**) Stacked bar plot showing the distribution of Con and CRKP samples across enterotypes E1 and E2.

As illustrated in Fig. 2, the gut microbiota composition in both Con and CRKP-infected individuals was primarily dominated by four major phyla: Firmicutes, Bacteroidota, Proteobacteria, and Actinobacteriota (Fig. 2A). At the family level, Enterobacteriaceae, Enterococcaceae, Lachnospiraceae, and Bacteroidaceae were the most abundant taxa across all samples (Fig. 2B). Genus-level analysis further revealed that *Enterococcus*, *Klebsiella*, *Escherichia-Shigella*, and *Bacteroides* were the predominant genera (Fig. 2C). PCoA based on genus-level profiles revealed a clear separation of microbial communities into two distinct enterotypes (Fig. 2D). Enterotype 1 (E1) was characterized by a high abundance of *Bacteroides* and *Escherichia-Shigella*, whereas Enterotype 2 (E2) was dominated by *Klebsiella* and *Enterococcus* (Fig. 2E). Notably, the distribution of enterotypes was strongly associated with the clinical status of the subjects. The majority of controls (94.7%, 36/38) were classified into the *Bacteroides*-associated E1 cluster, whereas most CRKP-infected patients (78.9%, 30/38) belonged to the *Klebsiella*/*Enterococcus*-dominated E2 (Fig. 2F). This marked shift in enterotype prevalence demonstrates a significant restructuring of the gut microbial ecosystem associated with CRKP infection.

**Fig 3:**
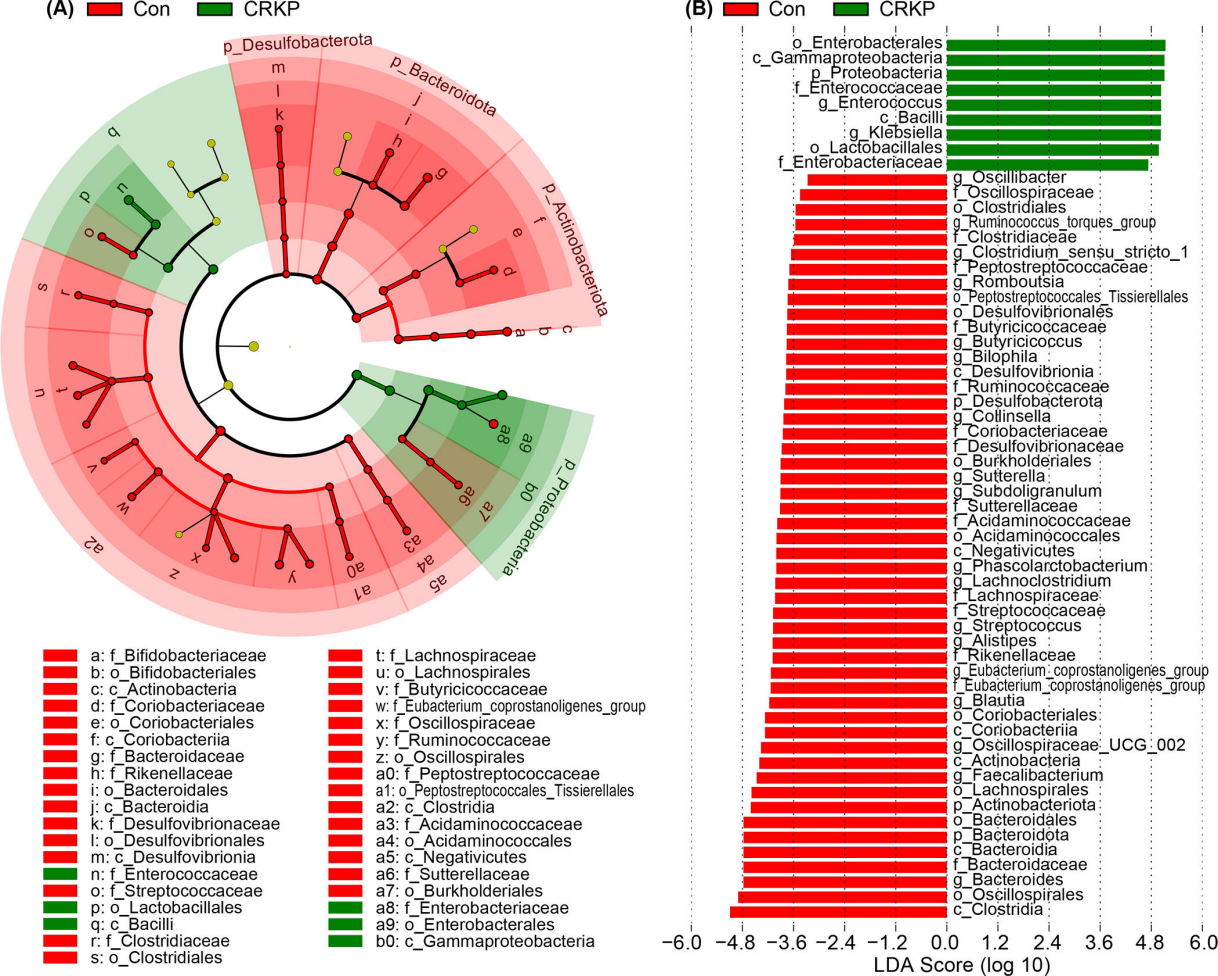
Differential microbial taxa identified by LEfSe analysis. (**A**) Cladogram illustrating the phylogenetic distribution of bacterial taxa that were significantly enriched in each group. Nodes are colored according to the group in which the taxon is most abundant: red for Con and blue for CRKP. Yellow nodes represent taxa without significant differences. The diagram displays the full taxonomic hierarchy from phylum to genus. (**B**) Histogram of LDA scores (log10) for the most discriminative features. Only taxa with an LDA score >3.0 and a significance of *P* < 0.05 (after FDR correction) are shown.

We then sought to identify the specific bacterial taxa responsible for the observed community-level shifts with LEfSe and MetaStats. As shown in the cladogram, the phylogenetic representation of taxa with differential abundance revealed significant alterations across multiple taxonomic levels (Fig. 3A). LEfSe analysis identified a clear dichotomy in enriched taxa between the two groups (LDA score > 3.0; Fig. 3B). The gut microbiota of CRKP-infected patients was significantly enriched in members of the phylum Proteobacteria, including the class Gammaproteobacteria, order Enterobacterales, and family Enterobacteriaceae. Within this family, the genera *Klebsiella* and *Enterococcus* were among the most strongly enriched taxa in the CRKP group. Conversely, the gut microbiota was characterized by a significant enrichment of beneficial and commensal taxa in the Con group. These included multiple members of the phylum Bacteroidota (e.g., class Bacteroidia, order Bacteroidales, family Bacteroidaceae, and genus *Bacteroides*) and the phylum Firmicutes. Notably, enriched Firmicutes taxa included the class Clostridia, order Oscillospirales, and several short-chain fatty acids (SCFAs) producers such as the genera *Faecalibacterium*, *Blautia*, and *Eubacterium*_*coprostanoligenes*_group. This targeted analysis confirms that CRKP infection is associated with a profound depletion of key commensal and health-associated bacteria, concomitant with a dramatic expansion of opportunistic pathogenic taxa, particularly *Klebsiella* and *Enterococcus*.

**Fig 4:**
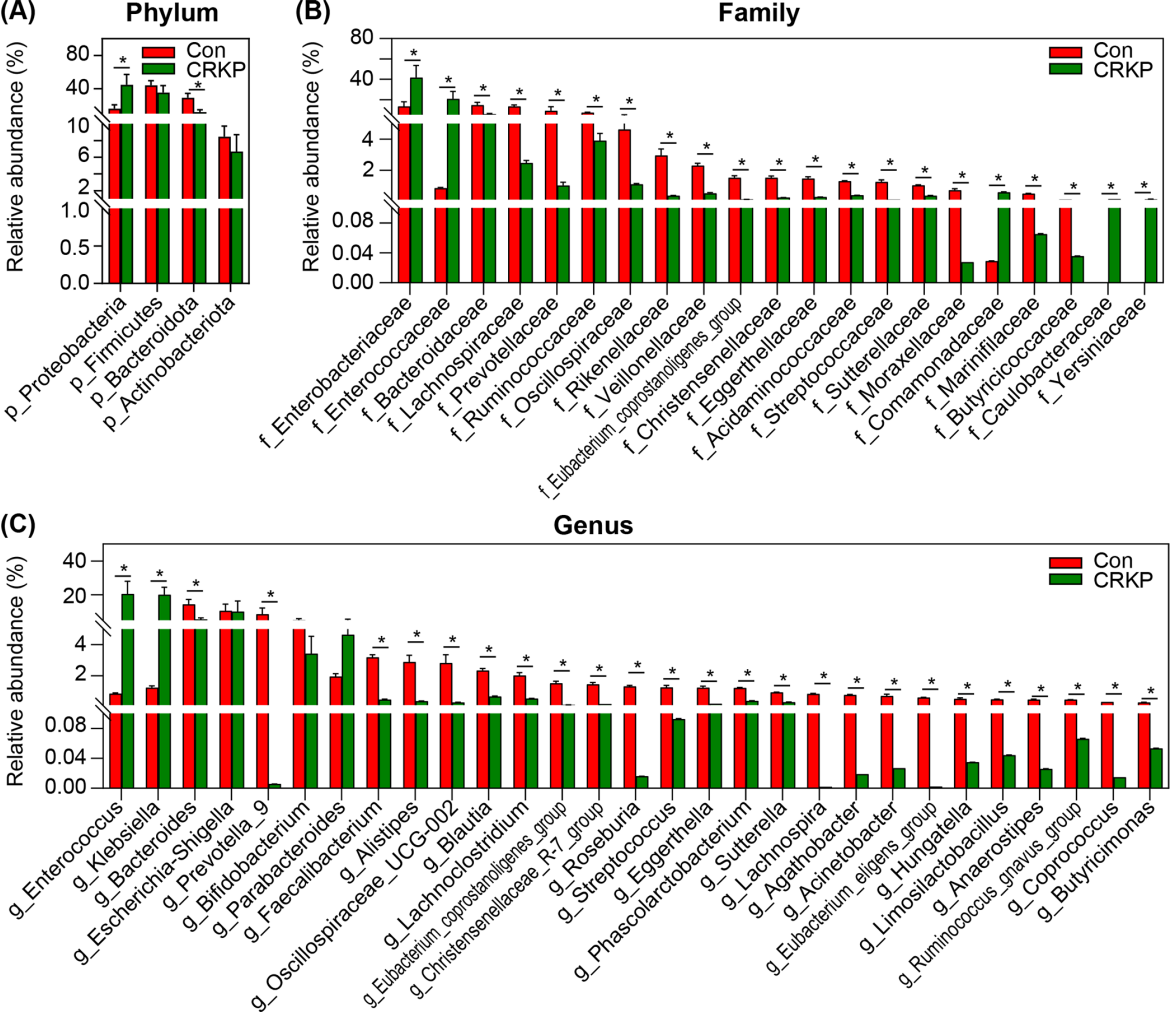
Differential abundance of bacterial taxa between CRKP-infected patients and controls (Con) as identified by MetaStats analysis. Relative abundances of significantly altered bacterial taxa at the phylum (**A**), family (**B**), and genus (**C**) levels are shown. Data are presented as mean ± standard deviation. Statistical significance was determined using the Mann–Whitney *U* test; *P < 0.05 compared with the Con group.

MetaStats 2.0 analysis further revealed significant differences in fecal microbiota between CRKP-infected patients and controls at the phylum, family, and genus levels. At the phylum level, CRKP patients exhibited a higher relative abundance of Proteobacteria and a reduction in Firmicutes and Bacteroidota (Fig. 4A). At the family level, Enterobacteriaceae and Enterococcaceae were significantly enriched in the CRKP group, while beneficial families like Lachnospiraceae, Ruminococcaceae, Bacteroidaceae, and Prevotellaceae were depleted (Fig. 4B). At the genus level, *Klebsiella*, *Enterococcus*, and *Escherichia-Shigella* were more abundant in CRKP patients, whereas Con had higher levels of beneficial genera such as *Bacteroides*, *Faecalibacterium*, *Prevotella_9*, *Blautia*, *Roseburia*, and *Eubacterium_coprostanoligenes*_group (Fig. 4C). These findings quantify the dysbiosis associated with CRKP infection, marked by an increase in pathobionts and a collapse of core health-associated taxa.

**Fig 5:**
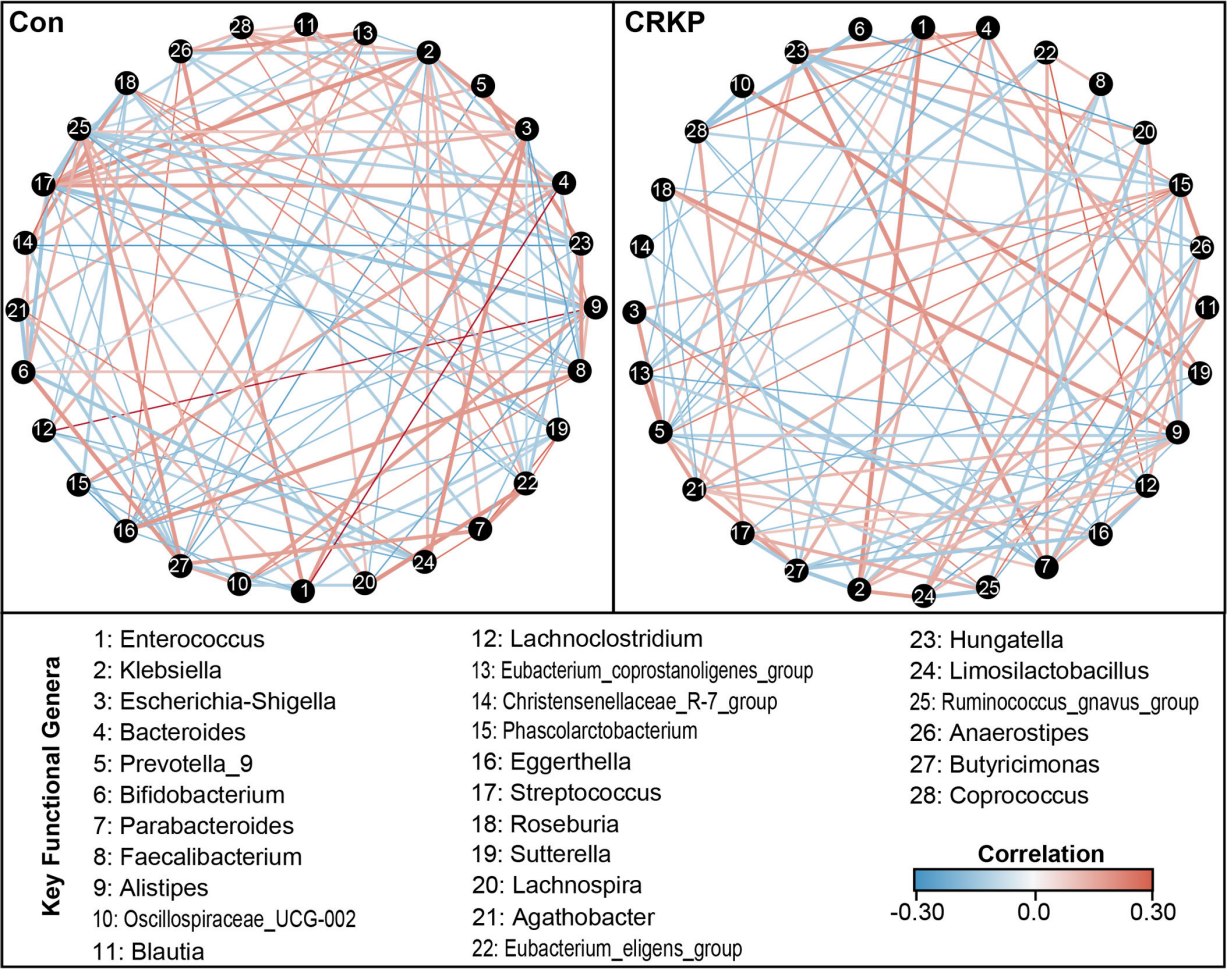
Microbial co-occurrence networks in fecal microbiota of controls (Con) and CRKP-infected patients. Correlation networks were constructed based on genus-level abundance data using the SparCC algorithm. Nodes represent 28 shared dominant bacterial genera, and edges represent significant correlations (red: positive; blue: negative). The network complexity and connectivity were markedly reduced in the CRKP group compared to the Con group, indicating disrupted microbial ecological interactions.

The fecal microbiota structure is shaped by dynamic microbial interactions. SparCC correlation network analysis revealed distinct microbial networks between the two groups (Fig. 5), with significant differences in topological properties (Table S1). In the Con group, the network contained 132 edges (significant correlations), with a high average degree (9.43) and network density (0.035). Positive correlations accounted for 76.5% of edges, primarily among beneficial genera such as *Bacteroides* (degree = 16), *Faecalibacterium* (degree = 14), and *Blautia* (degree = 12)—these core nodes exhibited high weighted degree centrality (8.92 ± 1.96), betweenness centrality (0.078 ± 0.019), and closeness centrality (0.34 ± 0.06), indicating a cooperative, stable microbial ecological structure. In contrast, the CRKP group’s network was drastically simplified: it contained only 58 edges, with a 56.1% reduction in average degree (4.14) and 51.4% reduction in network density (0.017) compared to Con. Negative correlations dominated (58.6% of edges), and core nodes shifted to pathobionts, including *Klebsiella* (degree = 10), *Enterococcus* (degree = 8), and *Escherichia-Shigella* (degree = 7). All centrality metrics were significantly lower in the CRKP network (weighted degree centrality: 4.85 ± 1.53; betweenness centrality: 0.029 ± 0.013; closeness centrality: 0.19 ± 0.04; all *P* < 0.001 vs. Con). Additionally, beneficial genera such as *Faecalibacterium* and *Roseburia* either disappeared from the network or showed weakened connectivity (degree <3). This destabilization of the microbial community structure and the dominance of pathogens highlights the microbial imbalance associated with CRKP infection, which may facilitate the persistence of multidrug-resistant organisms.

**Fig 6:**
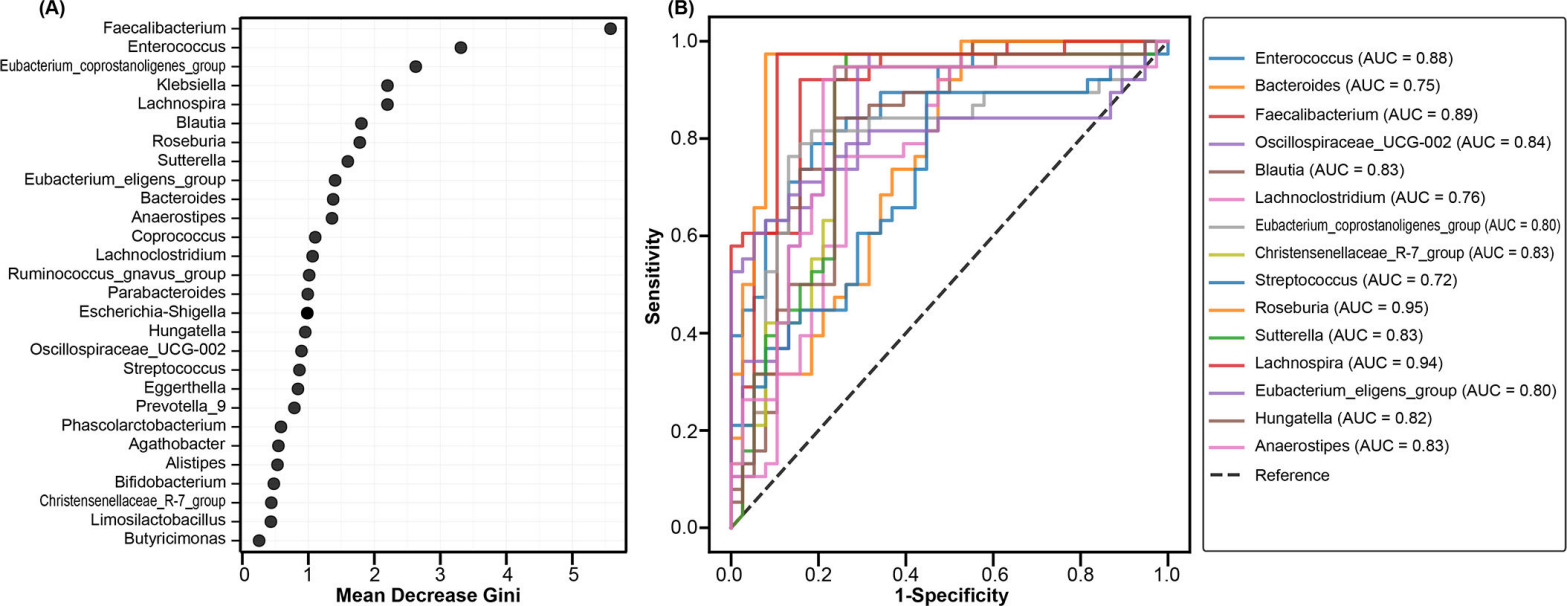
Discriminative power of gut microbial genera for identifying patients with CRKP infection. (**A**) Random Forest analysis showing the importance of bacterial genera based on the mean decrease Gini index. Higher values indicate a stronger contribution to classifying CRKP-infected patients versus controls (Con). (**B**) ROC curves illustrating the diagnostic performance of individual bacterial genera in distinguishing CRKP-infected patients from Con. The AUC value for each genus is indicated.

Random forest analysis was used to assess the potential of key bacterial taxa in distinguishing CRKP-infected patients from Con. Several genera, such as *Roseburia*, *Lachnospira*, *Faecalibacterium*, *Blautia*, and *Eubacterium_coprostanoligenes_*group, were more abundant in Con, while *Enterococcus* was identified as a key discriminative taxon for CRKP patients (Fig. 6A). The diagnostic potential of these taxa was evaluated using ROC curve analysis, revealing excellent discriminatory power for several genera, with AUC values above 0.8 (Fig. 6B). *Roseburia* (AUC = 0.95), *Lachnospira* (AUC = 0.94), and *Faecalibacterium* (AUC = 0.89) demonstrated high predictive capacity for Con, while *Enterococcus* (AUC = 0.88) was a strong predictor for CRKP infection. Other genera, including *Blautia*, *Sutterella*, *Christensenellaceae_*R-7*_group*, *Anaerostipes*, and *Oscillospiraceae_*UCG-002, also showed reliable discriminatory power (AUC = 0.83–0.84). These results indicate that specific bacterial genera, especially those involved in major fermenters/fermentation processes and gut health, can serve as non-invasive biomarkers for CRKP infection, positioning the gut microbiota as a potential diagnostic tool for detecting CRKP.

### Function shifts in the fecal microbiota of CRKP patients

**Fig 7:**
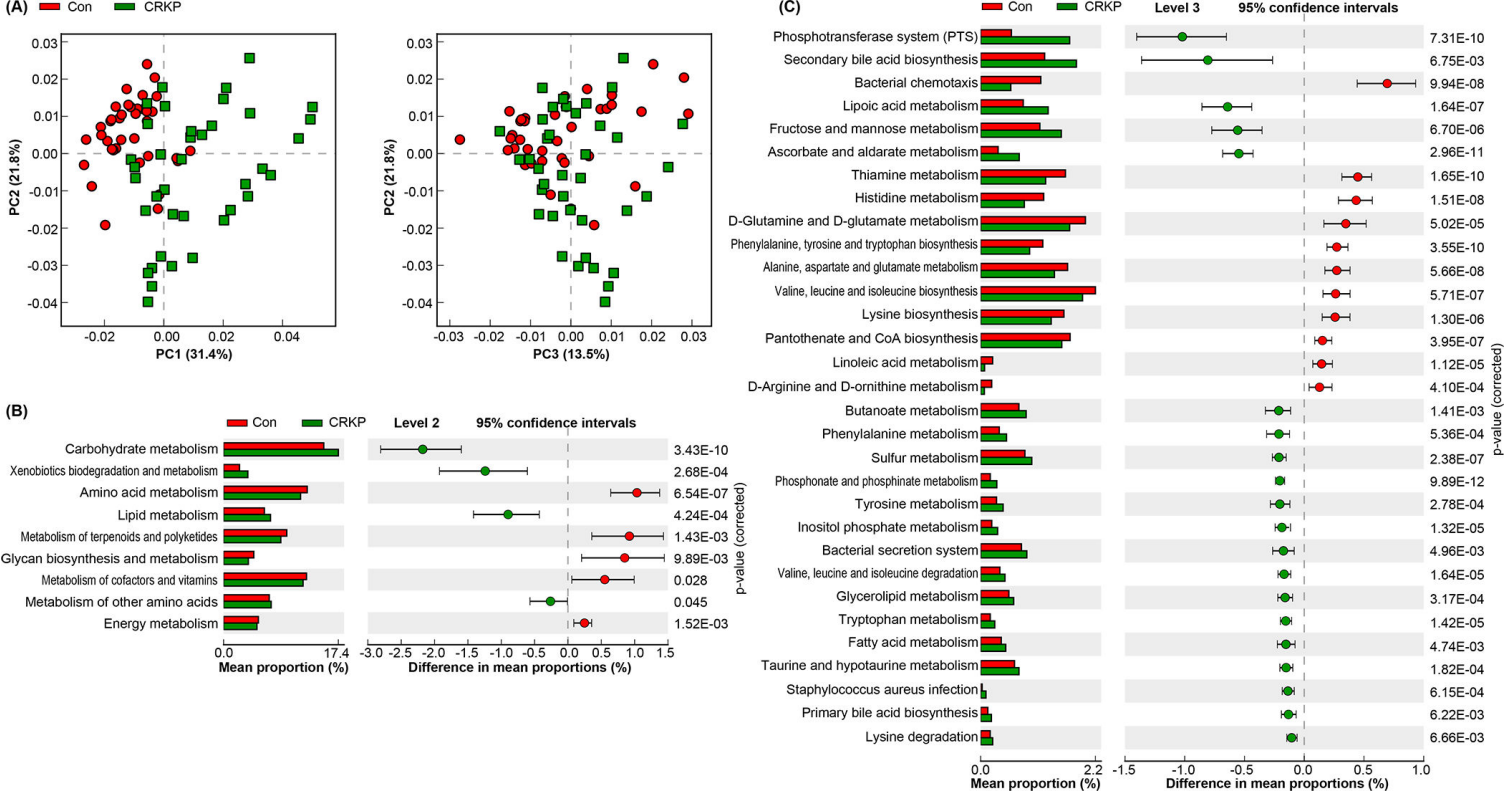
Predicted functional alterations in the gut microbiota of CRKP-infected patients compared to controls (Con) using PiCRUSt2. (**A**) PCA of predicted KEGG orthologs shows separation between groups. (**B**) Differentially abundant KEGG pathways at level 2. (**C**) Significantly altered metabolic and functional pathways at level 3. Statistical differences were assessed using Welch’s *t*-test followed by Benjamini-Hochberg FDR correction. Error bars represent 95% confidence intervals. Only pathways with a corrected *P*-value < 0.05 are displayed.

To investigate the functional consequences of the microbial shifts associated with CRKP infection, we performed a PiCRUSt2-based prediction of metagenomic functions (Fig. 7). PCA of the predicted KEGG pathways revealed a clear separation between the functional profiles of the Con and CRKP groups along the principal components, indicating profound functional dysbiosis (Fig. 7A). Comparative analysis of level 2 KEGG pathways showed that CRKP infection was associated with a significant decrease in crucial metabolic processes central to gut health. Most notably, pathways for carbohydrate metabolism were significantly depleted (*P* < 0.001). In contrast, pathways for xenobiotics biodegradation and metabolism were significantly enriched in the CRKP group (*P* < 0.001) (Fig. 7B). Further investigation at level 3 identified specific pathways that were differentially abundant. The CRKP group exhibited a significant reduction in vital biosynthetic and energy-harvesting pathways, including the phosphotransferase system (PTS) for sugar uptake, butanoate metabolism, and several pathways for the biosynthesis of essential amino acids (valine, leucine, and isoleucine biosynthesis; phenylalanine, tyrosine, and tryptophan biosynthesis) and vitamins (thiamine metabolism; pantothenate and CoA biosynthesis). Concurrently, there was a significant enrichment in pathways linked to pathogenicity and stress response, such as Bacterial secretion system, bacterial chemotaxis, and *Staphylococcus aureus* infection (Fig. 7C). Collectively, these functional predictions indicate that CRKP infection is associated with a pervasive disruption of the gut microbiome’s metabolic capacity, characterized by a loss of beneficial nutritional and metabolic functions and a concomitant rise in pathways potentially associated with virulence and xenobiotic resistance.

### Systemic immune dysregulation and microbial-host interplay in CRKP infection

**Fig 8:**
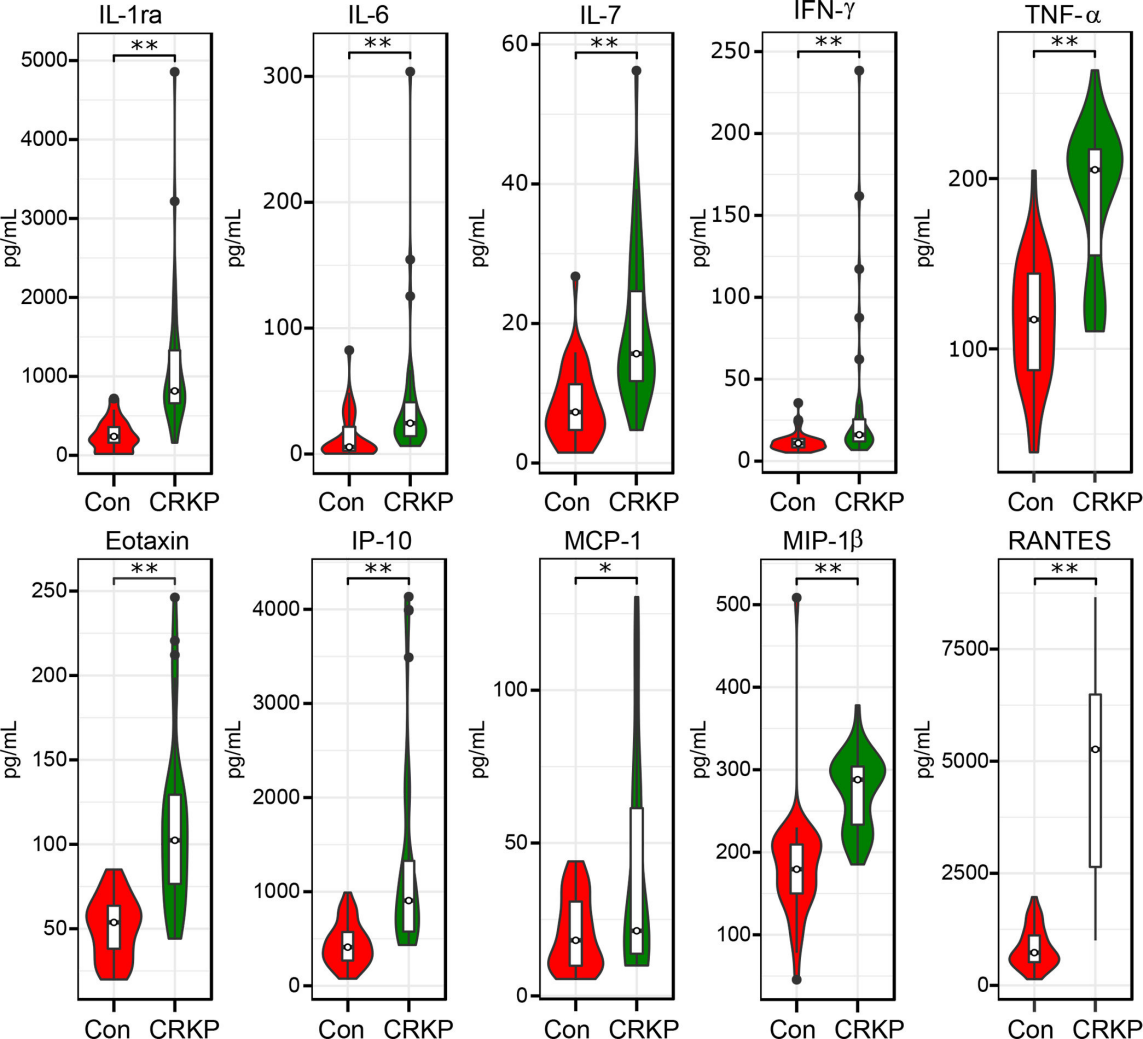
Systemic inflammatory cytokine and chemokine profiles in CRKP-infected patients and controls (Con). Serum levels of IL-1ra, IL-6, IL-7, IFN-γ, TNF-α, eotaxin, IP-10, MCP-1, MIP-1β, and RANTES were measured using a multiplex immunoassay. Concentrations of pro-inflammatory cytokines and chemokines were significantly elevated in the CRKP group compared to the Con group. Data are presented as scatter plots with mean ± standard deviation. *P < 0.05, **P < 0.01.

**Fig 9:**
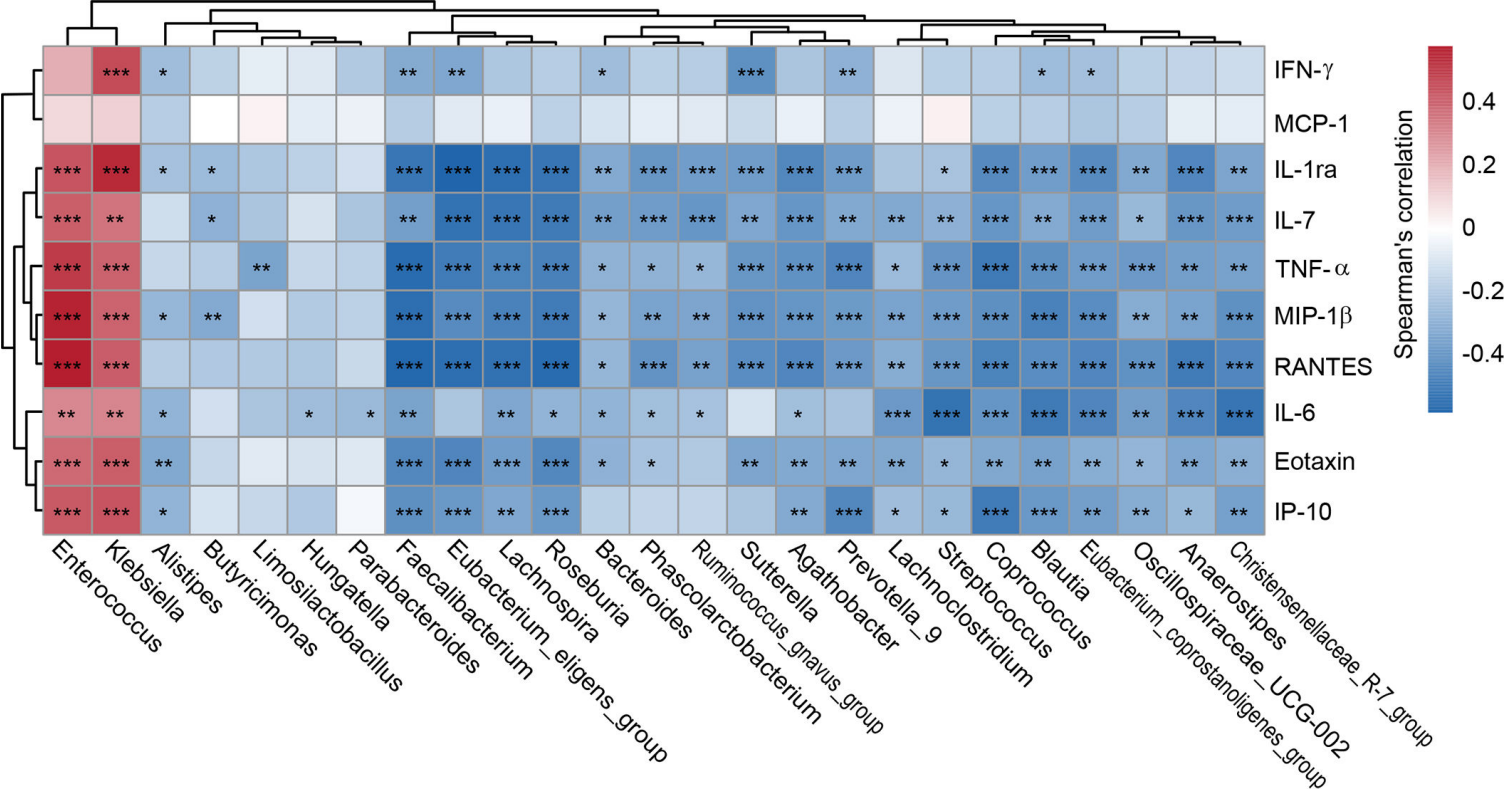
Correlation analysis between differentially abundant gut microbial genera and systemic immune markers in patients with CRKP infection. The heatmap illustrates Spearman’s rank correlation coefficients between the relative abundance of key bacterial genera and serum levels of cytokines and chemokines. Red indicates positive correlations, and blue indicates negative correlations. Statistical significance was determined using Spearman’s test with FDR correction; *P < 0.05, **P < 0.01, ***P < 0.001.

Analysis of systemic inflammatory cytokine profiles revealed significant immune dysregulation in CRKP-infected patients compared to Con. As shown in Fig. 8, serum concentrations of multiple pro-inflammatory cytokines and chemokines were significantly elevated in the CRKP group. Specifically, levels of IL-1ra, IL-6, IL-7, IFN-γ, and TNF-α, along with chemokines including eotaxin, IP-10, MCP-1, MIP-1β, and RANTES, were markedly increased in CRKP patients, reflecting a broad and potent activation of the host inflammatory response. Furthermore, integrative correlation analysis to elucidate the relationship between the altered gut microbiota and systemic inflammation revealed significant correlations between differentially abundant bacterial genera and key host cytokines (Fig. 9). The pathobionts *Klebsiella* and *Enterococcus*, which were enriched in CRKP patients, exhibited strong positive correlations with pro-inflammatory mediators such as IP-10, IL-6, and TNF-α. Conversely, beneficial SCFA producers that were depleted in the CRKP group, including *Faecalibacterium*, *Blautia*, and *Roseburia*, showed significant negative correlations with these same inflammatory markers. These results demonstrate that CRKP infection is characterized by a pronounced systemic inflammatory state and provide evidence for a direct link between gut microbiota dysbiosis and host immune dysfunction. The expansion of pro-inflammatory pathobionts coupled with the loss of beneficial commensals appears to drive a pro-inflammatory milieu, suggesting that the gut microbiome plays a key role in mediating systemic immune responses during CRKP infection.

## DISCUSSION

The gut microbiota, a complex and dynamic ecosystem, is integral to host physiology, particularly in the development and modulation of the immune system (30–32). It provides a critical first line of defense against invading pathogens, a concept known as CR, which is maintained through mechanisms like nutrient competition, secretion of antimicrobial peptides, and reinforcement of the gut barrier (33–36). Consequently, the stability and diversity of the gut microbiota are paramount for health (37). Disruption of this microbial homeostasis, or dysbiosis, is increasingly recognized as a key factor in the pathogenesis of numerous infectious and inflammatory diseases (32, 38). Critically ill patients are particularly susceptible to dysbiosis due to factors like antibiotic use, altered nutrition, and systemic inflammation, creating an environment permissive for the overgrowth of opportunistic pathogens (6, 39–43). This is especially relevant for multidrug-resistant organisms, such as CRKP, for which the colon serves as a primary reservoir (44, 45). A compromised microbiota loses its ability to resist colonization, increasing the risk of subsequent systemic infection and contributing to a cycle of inflammation and further dysbiosis, highlighting the critical importance of a balanced gut ecosystem in disease prevention and management (35, 46).

In this study, we provide a comprehensive characterization of the profound gut dysbiosis and associated systemic immune dysregulation in patients with active CRKP infection. Our finding of significantly reduced microbial alpha-diversity in CRKP patients aligns with observations in other critical illnesses and infections, where a loss of microbial richness and evenness is a common hallmark of a disturbed ecosystem (47, 48). A key and novel finding of our work is the dramatic shift in enterotype classification. While healthy individuals predominantly harbored a *Bacteroides*-dominated enterotype (E1), a community structure associated with metabolic health, the vast majority of CRKP patients transitioned to a *Klebsiella*/*Enterococcus*-dominated enterotype (E2). This stark dichotomy differs from more subtle shifts reported in other conditions and points to a near-complete restructuring of the gut community driven by the pathogenic expansion of *Klebsiella*. This observation suggests that active CRKP infection does not merely alter the gut environment but actively remodels it to favor its own dominance, potentially by outcompeting commensal microbes for resources or through direct antagonism (36, 45).

Our analysis identified specific bacterial taxa that drive this community-wide shift, providing functional insights into the dysbiotic state. The dramatic enrichment of *Klebsiella* and *Enterococcus* in CRKP patients is particularly concerning. *K. pneumoniae* itself is a pathobiont whose gastrointestinal colonization is a major risk factor for subsequent infection (45). *Enterococcus* species, often considered commensals, are also notorious opportunistic pathogens that can thrive in the dysbiotic gut of hospitalized patients and are known for their intrinsic and acquired antibiotic resistance (49, 50). The synergy between these two pathogens is increasingly recognized; they can coexist and potentially facilitate each other’s persistence and virulence (51). Concurrently, we observed a profound depletion of beneficial, butyrate-producing commensals, including *Faecalibacterium*, *Blautia*, and *Roseburia*. These bacteria are crucial for maintaining gut health. Butyrate, their primary metabolic product, serves as the main energy source for colonocytes, strengthens the epithelial barrier, and exerts potent anti-inflammatory effects (36, 52, 53). The loss of these keystone species cripples the microbiota’s ability to provide CR. For instance, a reduction in butyrate-producing bacteria leads to increased oxygen availability at the gut epithelial surface, which favors the growth of facultative anaerobes like *Klebsiella* over obligate anaerobes, further fueling the cycle of dysbiosis (52). Thus, the gut environment in CRKP patients is characterized by a dual assault: the expansion of pro-inflammatory pathobionts and the collapse of the protective commensal community.

These compositional shifts translated into a profoundly altered functional landscape, as predicted by our metagenomic analysis. The depletion of pathways for butanoate metabolism and PTS in CRKP patients directly reflects the loss of metabolically versatile commensals. This functional impairment signifies a reduced capacity for energy harvest from dietary fibers and for producing butyrate, a metabolite critical for maintaining epithelial barrier integrity and immune homeostasis (54, 55). Furthermore, the enrichment of pathways related to virulence, such as bacterial secretion systems and chemotaxis, suggests a functional pivot toward a more pathogenic community profile. Bacterial secretion systems, particularly Type III and Type VI, are essential tools used by pathogens like *K. pneumoniae* to inject effector proteins into host cells, subvert immune responses, and kill competing bacteria (56–58). Simultaneously, the upregulation of xenobiotic biodegradation and secondary bile acid biosynthesis pathways points to a microbial community adapted for survival in a hostile, medically treated environment. An enhanced ability to metabolize xenobiotics may contribute to antibiotic tolerance, while altered bile acid pools can selectively promote the growth of resistant pathogens like CRKP, which are more tolerant to their antimicrobial effects than many commensals (59, 60). Collectively, the predicted functional profile of the CRKP gut microbiome is one of a disabled commensal community with impaired metabolic and nutritional functions, alongside an empowered pathogenic consortium armed with enhanced virulence and resistance mechanisms.

This study further establishes a strong link between the observed gut dysbiosis and the systemic hyper-inflammatory state characteristic of severe CRKP infection. The elevated levels of pro-inflammatory cytokines (IL-6, TNF-α, IFN-γ) and chemokines (IP-10, MCP-1) in CRKP patients are indicative of a robust and potentially dysregulated host immune response (47). Crucially, our correlation analysis provides evidence that the gut microbiota is not a passive bystander but an active participant in modulating this systemic response. The positive correlation between pathobionts like *Klebsiella* and *Enterococcus* and pro-inflammatory mediators such as IL-6 and TNF-α suggests that the overgrowth of these bacteria in the gut may directly contribute to systemic inflammation, possibly through the translocation of microbial products like lipopolysaccharide across a compromised gut barrier (38, 48). Conversely, the strong negative correlation between beneficial butyrate producers (*Faecalibacterium*, *Blautia*, *Roseburia*) and these same inflammatory markers underscores their immunomodulatory role. Metabolites produced by these commensals, particularly SCFAs like butyrate, are known to regulate immune cell function, promote the differentiation of anti-inflammatory regulatory T cells (Tregs), and dampen inflammatory responses (61–64). For example, *F. prausnitzii* has been shown to exert potent anti-inflammatory effects, partly through secreted metabolites that block NF-κB activation (65–67). Therefore, the depletion of these bacteria in CRKP patients likely results in a loss of these crucial anti-inflammatory signals, leaving the host’s pro-inflammatory response unchecked and contributing to the systemic immune dysfunction observed (68).

While this study provides significant insights into the host-microbe dynamics during CRKP infection, certain limitations must be acknowledged. First, the cross-sectional case–control design allows identification of associations but precludes causal inference regarding the relationship between gut dysbiosis and systemic inflammation. It remains unclear whether microbial dysbiosis drives immune dysfunction, whether systemic inflammation induced by CRKP infection disrupts gut homeostasis, or whether both processes act synergistically. Second, this single-center study may limit the generalizability of our findings across different patient populations and geographic regions. Third, although the sample size was statistically sufficient to detect major shifts in the gut microbiota composition, larger multicenter cohorts are still required to validate these observations. Additionally, due to constraints in sample size and the high prevalence of multi-site CRKP colonization/infection among critically ill patients, we were unable to perform stratified analyses based on the primary source of CRKP infection, which may have revealed infection site-specific microbial patterns. Fourth, while the gastrointestinal tract hosts segment-specific microbial communities (e.g., distinct taxa in the small intestine, proximal colon, and distal colon/rectum), our use of fecal samples—primarily representative of distal colonic/rectal luminal microbiota—as a surrogate for the entire gut microbiota may not fully capture microbial composition across all gut segments. Finally, this study did not investigate the underlying mechanisms of these interactions, such as by measuring gut barrier integrity or microbial metabolite levels directly, nor did it explore potential interventions. Future research should employ longitudinal study designs to track the dynamic interplay between the microbiota, the immune system, and CRKP infection over time, from colonization to the resolution of infection, which would help elucidate causal relationships.

### Conclusion

In summary, this study demonstrates that systemic CRKP infection is associated with a profound gut dysbiosis, characterized by a loss of microbial diversity, a collapse of beneficial butyrate-producing commensals, and a dramatic expansion of opportunistic pathobionts, including *Klebsiella* and *Enterococcus*. This microbial imbalance is tightly linked to a dysregulated systemic immune response, marked by excessive pro-inflammatory cytokine production. The strong correlations between specific microbial signatures and host inflammatory markers highlight the critical role of the gut-host immune axis in the pathophysiology of CRKP infection. These findings suggest that the gut microbiota could serve as both a biomarker for disease severity and a therapeutic target. Interventions aimed at restoring microbial homeostasis, such as the use of targeted probiotics, prebiotics, or FMT, may represent promising adjunctive strategies to mitigate immune dysfunction and improve clinical outcomes in patients suffering from this challenging infection.

Future investigations using gnotobiotic or antibiotic-treated murine models will be critical to delineate causal links between gut dysbiosis and CRKP susceptibility. Experimental interventions such as microbiota transfer from healthy or CRKP-infected donors, or administration of defined microbial consortia, will help clarify whether targeted microbiota modulation can enhance CRKP clearance and restore immune equilibrium. These mechanistic studies will be essential to validate the gut microbiota as both a biomarker and a therapeutic target in CRKP infection.

## Data Availability

The sequence data from this study are deposited in the GenBank Sequence Read Archive with the accession number PRJNA1265392.

## SUPPLEMENTAL MATERIAL

Table S1 spectrum.02975-25-s0001.docx Quantitative indicators of SparCC-derived microbial co-occurrence networks.

ASM does not own the copyrights to Supplemental Material that may be linked to, or accessed through, an article. The authors have granted ASM a non-exclusive, world-wide license to publish the Supplemental Material files. Please contact the corresponding author directly for reuse.
