# Supplementary material for: Impact of carbapenem-resistant Klebsiella pneumoniae infection on gut microbiota and host immunity: a case-control study
Source: Microbiol Spectr. 2025 Nov 20;14(1):e02975-25. doi: 10.1128/spectrum.02975-25 (PMC12772321; doi:10.1128/spectrum.02975-25)
Supplement: Table S1 — Quantitative indicators of SparCC-derived microbial co-occurrence networks. [file spectrum.02975-25-s0001.docx]

**Table S1 Quantitative indicators of SparCC-derived microbial co-occurrence networks**

| **Network Metric** | **Con** | **CRKP** | **p-value** |
| --- | --- | --- | --- |
| Number of Nodes | 28 | 28 |  |
| Number of Significant Edges | 132 | 58 |  |
| Positive Edges | 76.5% (101/132) | 41.4% (24/58) | <0.001 |
| Negative Edges | 23.5% (31/132) | 58.6% (34/58) | <0.001 |
| Average Degree | 9.43 | 4.14 | <0.001 |
| Network Density | 0.035 | 0.015 | <0.001 |
| Core Nodes | *Bacteroides* (degree = 16) | *Klebsiella* degree = 10) | - |
|  | *Faecalibacterium* (degree = 14) | *Enterococcus* degree = 8) |  |
|  | *Blautia* (degree = 12) | *Escherichia-Shigella* degree = 7) |  |
| Weighted Degree Centrality | 8.92 ± 1.96 | 4.85 ± 1.53 | <0.001 |
| Betweenness Centrality | 0.078 ± 0.019 | 0.029 ± 0.013 | <0.001 |
| Closeness Centrality | 0.34 ± 0.06 | 0.19 ± 0.04 | <0.001 |
